# Investigation of TaC and TiC for Particle Strengthening of Co-Re-Based Alloys

**DOI:** 10.3390/ma16237297

**Published:** 2023-11-23

**Authors:** Eugen Seif, Joachim Rösler, Jonas Werner, Thomas E. Weirich, Joachim Mayer

**Affiliations:** 1 Institute for Materials Science, Technische Universität Braunschweig, Langer Kamp 8, 38106 Braunschweig, Germany; e.seif@tu-bs.de; 2 Central Facility for Electron Microscopy (GFE), RWTH Aachen University, Ahornstraße 55, 52074 Aachen, Germany; weirich@gfe.rwth-aachen.de (T.E.W.); mayer@gfe.rwth-aachen.de (J.M.)

**Keywords:** high-temperature alloys, Co-Re alloys, TaC, TiC, creep, scanning transmission electron microscopy, ASTAR, scanning precession electron diffraction

## Abstract

Cobalt-Rhenium (Co-Re)-based alloys are currently investigated as potential high-temperature materials with melting temperatures beyond those of nickel-based superalloys. Their attraction stems from the binary Co-Re phase diagram, exhibiting complete miscibility between Co and Re, whereby the melting temperature steadily increases with the Re-content. Thus, depending on the Re-content, one can tune the melting temperature between that of pure Co (1495 °C) and that of pure Re (3186 °C). Current investigations focus on Re-contents of about 15 at.%, which makes melting with standard equipment still feasible. In addition to solid solution strengthening due to the mixture of Co- and Re-atoms, particle strengthening by tantalum carbide (TaC) and titanium carbide (TiC) precipitates turned out to be promising in recent studies. Yet, it is currently unclear which of the two particle types is the best choice for high temperature applications nor has the strengthening mechanism associated with the monocarbide (MC)-precipitates been elucidated. To address these issues, we perform compression tests at ambient and elevated temperatures on the particle-free base material containing 15 at.% of rhenium (Re), 5 at.% of chromium (Cr) and cobalt (Co) as balance (Co-15Re-5Cr), as well as on TaC- and TiC-containing variants. Additionally, transmission electron microscopy is used to analyze the shape of the precipitates and their orientation relationship to the matrix. Based on these investigations, we show that TiC and TaC are equally suited for precipitation strengthening of Co-Re-based alloys and identify climb over the elongated particles as a rate controlling particle strengthening mechanism at elevated temperatures. Furthermore, we show that the Re-atoms are remarkably strong obstacles to dislocation motion, which are overcome by thermal activation at elevated temperatures.

## 1. Introduction

Co-Re-based alloys pose a new class of metallic high-temperature materials. These alloys gain their high temperature strength from solid solution hardening of Co and Re [1], as well as particle strengthening with precipitates such as the intermetallic chromium rhenium phase Cr_2_Re_3_ [2], chromium carbide Cr_23_C_6_ and TaC [3]. However, only TaC offers great potential for high-temperature applications because Cr_23_C_6_ dissolves at approx. 1000 °C [4], while Cr_2_Re_3_ exhibits pronounced coarsening behavior at 1100 °C [2]. In contrast, TaC precipitates as nano-sized particles and is thermally stable up to approx. 1300 °C [4,5,6]. Co-Re-based alloys, up to an amount of approx. 25 at.% Re, are allotropic and feature just below the solidus temperature either a single phase region of the face centered cubic fcc-Co high temperature phase for up to 17 at.% Re or for higher Re-contents a two-phase region of fcc-Co and the hexagonal close-packed hcp-Co low temperature phase [7]. It is possible that some of the fcc-Co is metastably retained upon cooling, which causes a variety of disadvantages in creep application observed for TaC-strengthened Co-Re alloys. TaC dissolves in fcc-Co (which is utilized for the dissolution of TaC during the solution heat treatment at 1450 °C), but remains stable in hcp-Co thereby limiting the maximum creep temperature to the stability range of the hcp-Co phase. Moreover, fcc-Co is considerably softer than hcp-Co [8] and creep processes tend to occur faster for Co-Re alloys with more retained fcc-Co [9]. Therefore, it is necessary to prevent the formation of retained fcc-Co, which can be accomplished by either the addition of Cr or reducing the free amount of the fcc-stabilizing and MC-forming elements tantalum (Ta) and carbon (C) via a stochiometric composition (e.g., Co-Re-Cr-xTa-xC) [10]. Recently, the research focus was set on TaC, TiC and hafnium carbide (HfC) precipitates in Co-Re-Cr, where the latter, unlike TaC and TiC, yielded a distribution of coarse HfC and intermetallic Co-Hf phases. Only an insignificant amount of HfC precipitated as nano-sized particles [11]. For this reason, this study focuses on TiC and TaC, investigating their morphology and effect on mechanical behavior via compression tests at room temperature, as well as strain-rate controlled creep tests at 800 °C and 900 °C. To ensure a single-phase fcc state during solution heat treatment and a single-phase hcp state at ambient and elevated temperatures, Co-15Re-5Cr was used as basis (all mentioned compositions throughout this work are stated in at.%). For the particle strengthened materials, Co-15Re-5Cr-1.8Ta-1.8C and Co-15Re-1.8Ti-1.8C were selected as composition because prior work [11] showed that 1.8% of the MC-forming elements is close to the solubility limit at temperatures around 1450 °C. Furthermore, the orientation relationship for these MC carbides is determined with precession-enhanced selected area electron diffraction (P-SAED) and scanning precession electron diffraction (SPED). Finally, transmission electron microscopy (TEM) images of creep-deformed specimens provide clear evidence of particle–dislocation interaction for both TaC and TiC. 

## 2. Materials and Methods

Casting of Co-15Re-5Cr-1.8Ta-1.8C, Co-15Re-5Cr-1.8Ti-1.8C and Co-15Re-5Cr was conducted in a two-step melting process. Firstly, the master alloy of Co-20Re was cast in a vacuum induction furnace at VDM Metals International GmbH in Werdohl (Germany). Secondly, the respective monocarbide-forming elements were added to the master alloy to obtain the targeted composition. In the case of Co-15Re-5Cr, this was achieved by diluting the high Re-content of the master alloy with additional amounts of Co and Cr. All alloys were cast by vacuum arc melting (VAM) in a PINK vacuum arc furnace (Wertheim, Germany) as rods with a diameter of 13 mm and a height of approx. 100 mm and received the same solution heat treatment (ST) of 1350 °C/5 h + 1400 °C/5 h + 1450 °C/5 h with subsequent argon (Ar) gas quenching in a Linn High Therm KKH-200/200/350/1600 Moly vacuum furnace (Eschenfelden, Germany). Subsequently, an aging heat treatment either at 900 °C or 1100 °C for 15 h was performed for the MC-strengthened alloys in a vacuum furnace. Although Co-15Re-5Cr is a pure solid solution alloy, the authors also conducted an additional heat treatment at 900 °C for 15 h after solution heat treatment and gas quenching in order to remove residual stresses and adjust the grain size for better comparability with TaC- and TiC-strengthened Co-15Re-5Cr after aging. The MC-strengthened alloys are denoted as TaC9, TaC11, TiC9, TiC11 where the letters refer to the MC-type (TaC and TiC) and the following number to the aging treatment (9 = 900 °C/15 h and 11 =1100 °C/15 h). The MC-free alloy is denoted as Ref9 since it is used as a reference for the strengthening contribution of the solid solution matrix, whereby the number refers again to the aging temperature, as aforementioned. 

The scanning electron microscope (SEM) images of the microstructures were taken with a Helios NanoLab 650 from FEI (Eindhoven, The Netherlands). The orientation relationship of needle-shaped TaC and TiC precipitates was analyzed with transmission electron microscopy (TEM). For that purpose, lamellae of TaC9 and TiC9 were prepared by focused ion beam (FIB) machining with a FEI–Strata 400 S dual-beam FIB (Eindhoven, Netherlands). TEM investigation was conducted using a 200 kV JEOL–JEM F200 machine from JEOL Ltd. (Tokyo, Japan). In-depth analysis of the orientation relationship of precipitates and matrix in the TiC9 lamella was carried out with SPED and subsequent automated crystal orientation mapping (ACOM) [12] achieved with the Nanomegas–ASTAR device and software suite (Brussels, Belgium). Data processing with ACOM was based on diffraction pattern templates of CoRe and TiC crystallographic information file (CIF) data [13,14,15] from ICSD (Karlsruhe, Germany). Cross-referential determination of the orientation relationship was carried out by precession-technique-enhanced selected area electron diffraction (P-SAED) analysis and precipitate morphology confirmation was performed with serial tilt imaging using a Gatan–OneView complementary metal-oxide-semiconductor (CMOS) camera (Pleasonton, CA, USA).

For the compression tests at room temperature and compression creep tests on TaC9, TaC11, TiC9, TiC11 and Ref9, specimens were wire-cut into cylinders with a diameter of 10 mm and a height of 17 mm. The compression tests were conducted on a Zwick Roell universal testing machine BZ1-MMRM200.ST01 (Ulm, Germany) with a preload of 100 MPa to put all components of the load line under compression prior to the test. The strain was measured by a GOM ARAMIS 4M system (Braunschweig, Germany). The creep experiments were performed in an Ar atmosphere at 500 mbar ambient pressure after several steps of evacuation and flushing with Ar to minimize the oxygen content within the vacuum chamber. Throughout the creep experiment, the specimen was heated by induction heating. Single crystalline CMSX4 plates were placed on the top and bottom of the specimen so that the magnetic field couples with both plates and the specimen; thus, generating a homogeneous heat distribution across the entire specimen. The temperature was monitored with three thermocouples on the lateral surface in the middle and close to both faces of the specimen. The displacement was measured with a ceramic high-temperature extensometer. Multi-step creep experiments, varying the strain rate within one test, were carried out at 800 °C and 900 °C. Considering the binary phase diagram of Co-Re at 15 at.% of Re, the melting temperature T_m_ of approx. 1560 °C (1833 K) suggests that the creep experiments were carried out at approx. 58% and 64% of T_m_, respectively. The strain rate was held constant at 10^−6^ s^−1^ (exactly: 1.67 × 10^−6^ s^−1^), 10^−5^ s^−1^ and 10^−4^ s^−1^ within the strain steps of 0–2%, 2–4% and 4–6%, respectively. An additional creep experiment was performed at 800 °C for TaC9 and TiC9 at stresses of 500 MPa and 450 MPa, respectively. Upon reaching the secondary creep state for TaC9 and TiC9 at approx. a strain of 2.4% and 0.6%, respectively, the induction heating was switched off while the stress was kept constant in an attempt to freeze the dislocation configuration as it was present during creep for further TEM investigation.

## 3. Results

### 3.1. Precipitation, Morphology and Orientation Relationship

**Figure 1:**
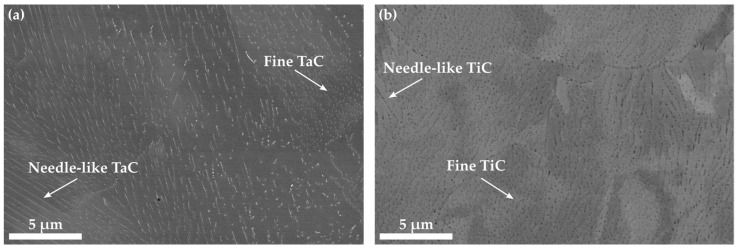
Back-scattered electron images illustrating the microstructure of TaC9 in (**a**) and TiC9 in (**b**) with the precipitation of nano-sized TaC and TiC, respectively [11].

A prior study on the precipitation behavior of TaC and TiC in Co-Re-Cr was conducted in [11], from which the results in Figure 1 stem. For a holistic presentation of the precipitation behavior, the authors decided to provide scanning electron images of the microstructure in addition to the new images from TEM. Figure 1 displays the microstructure of TaC9 and TiC9. Both microstructures are characterized by the distribution of fine and elongated particles on the nano-scale. The precipitation is homogeneous so that grain boundaries do not alter the shape or size of the particles. Furthermore, a distinct fluctuation of background color within the matrix is visible, which probably originates from the material contrast of locally varying Re contents within the Co-Re-Cr matrix.

**Figure 2:**
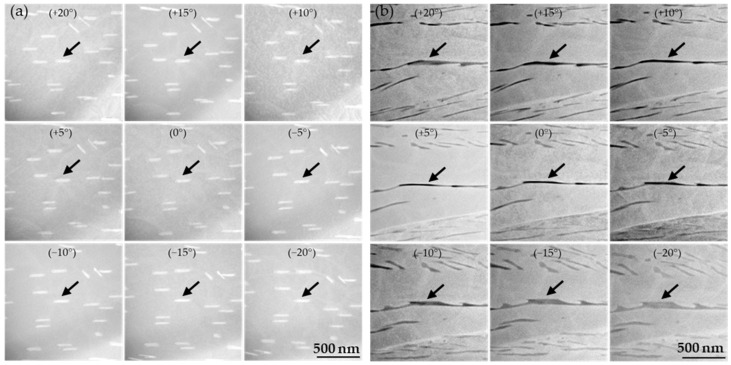
A series of tilted STEM Z-contrast ADF images (tilting range: +20° to −20°) from the lamella of crept TaC9 and TiC9 depicting the morphologies of TaC and TiC in (**a**) and (**b**), respectively. The unchanging cross-section of TaC suggests a cylindrical shape, whereas the cross-section of TiC appears to be rectangular due to particle broadening upon tilting. The arrows mark one specific carbide for better visual tracking in the series of tilted images.

The morphology of each precipitate phase can be deduced from the series of tilted scanning transmission electron microscopy (STEM) mean atomic number contrast-dominated annular dark field (Z-contrast ADF) images shown in Figure 2a,b for TaC9 and TiC9, respectively. Both TaC and TiC feature an elongated shape where the lateral axis of the precipitates is longer than the perpendicular one.

Taking an edge-on look at one specific TaC particle (marked by arrows) and tilting the TEM image from +20° to −20° (rotation around the lateral axis), it becomes evident that the length of the perpendicular axis of TaC does not change. This suggests a circular cross-section of the TaC particles. Contrary to TaC, TiC shows an increase in length on the perpendicular axis, indicating a rectangular cross-section, see, for example, the arrows at tilt angles of 0° and −20 °C. However, the particle length into the depth of the lamella is not sufficient to consider TiC plate-like, making TiC rather bar-like. Both the average particle radius of TaC and the average length of the perpendicular axis of TiC in the 0° tilted image are approx. 20 nm.

**Figure 3:**
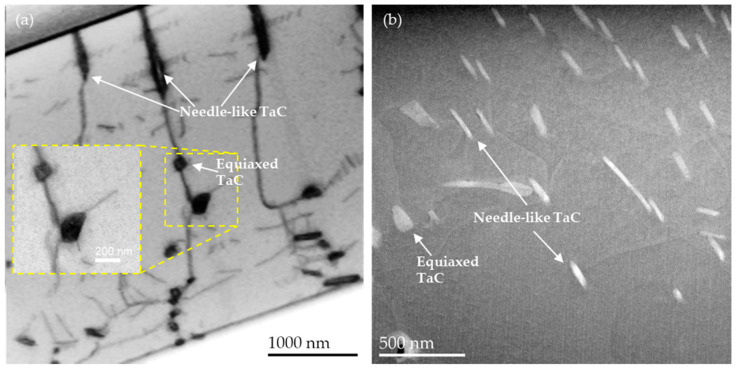
ASTAR virtual bright field image in (**a**) and STEM Z-contrast ADF image in (**b**) depicting a selection of elongated (needle-like) and equiaxed TaC in various lamellae of TaC9.

Even though it appears that the majority of the particles have an elongated shape as depicted in Figure 2 it should be noted that also roughly equiaxed TaC particles can be found, see Figure 3 and [16,17].

**Figure 4:**
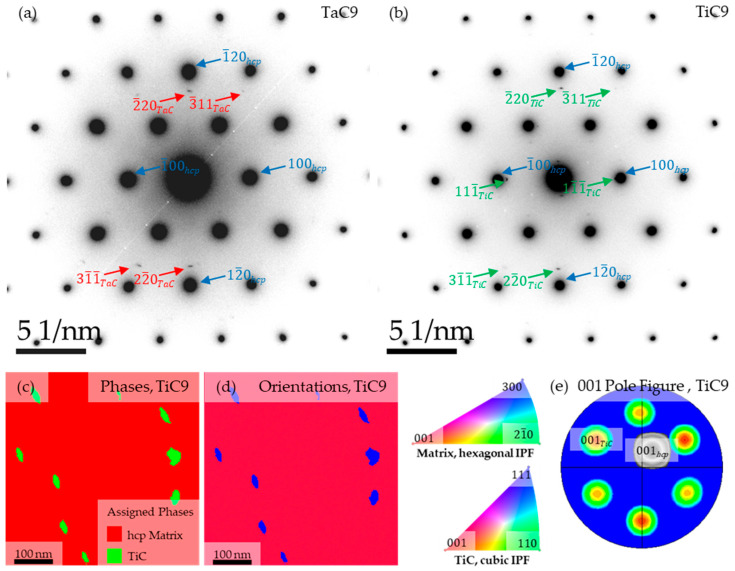
P-SAED patterns obtained from the [001]_hcp_-oriented matrix and needle-like precipitates as well as complementary ACOM phase and orientation analysis of radial precipitate cross sections with corresponding IPF-color codes for the orientation map and {001}-pole figure. The diffraction pattern of the matrix (reflections expemplarily marked blue) is depicted with reflection positions of TaC-precipitates (marked red) in (**a**) and TiC-precipitates (green) in (**b**). ACOM analysis (**c**–**e**) reveals an approximate orientation relationship of ($\bar{1}$20)_hcp-Co_||($\bar{1}$10)_TiC_, [00$\bar{1}$]_hcp-Co_||[112]_TiC_ as is further indicated by the (001)_hcp_ pole figure. The precipitate reflection positions observed in (**b**) coincide with low order bragg reflections expected for the [112]_TiC_ zone axis orientation hence supporting the orientation relationship found with ACOM. As equivalent reflections are observed in (**a**), it is inferred that needle-like precipitates in TaC9 and TiC9 exhibit the same orientation relationship.

Figure 4 shows P-SAED patterns of the hcp-Co matrix in [001]_hcp_ zone axis orientation superimposed with distinct precipitate reflections on positions characteristic for TaC and TiC unit cell geometry (space group #225: $Fm\bar{3}m$) according to reported CIF data [14,15], as well as ACOM maps and corresponding [100] pole figure for needle-like TiC precipitates. 

Precipitate reflections visible in the P-SAED pattern of the TiC9 sample are in agreement with the orientation relationship extracted with ACOM from SPED, suggesting an approximate orientation relationship of ($\bar{1}20$)_hcp-Co_||($\bar{1}10$)_TiC_, [$00\bar{1}$]_hcp-Co_||[112]_TiC_. Since the P-SAED patterns of TaC9 and TiC9 share common precipitate reflection positions, it can be surmised that the orientation relationship of needle-like precipitates in TaC9 is similar to the measured relationship in TiC9.

### 3.2. Compression Tests at Ambient and Elevated Temperatures, Particle–Dislocation Interaction

**Figure 5:**
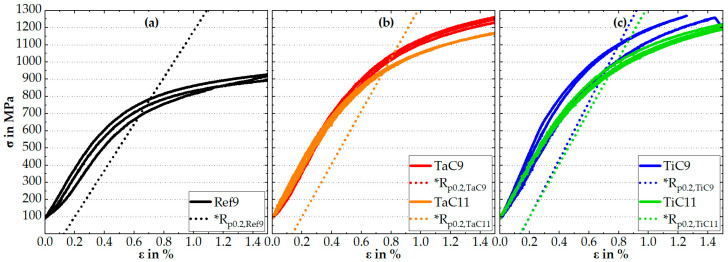
Stress–strain curves from the compression tests (with a preload of 100 MPa) at room temperature for Ref9 in (**a**), for TaC9 and TaC11 in (**b**), as well as for TiC9 and TiC11 in (**c**). The auxiliary lines to determine the yield strength *R_p0.2_ are also shown.

Figure 5 illustrates stress–strain curves from the compression tests at room temperature for Ref9, TaC9, TaC11, TiC9 and TiC11, while Table 1 gives an overview of the average value of the compressive Young’s modulus *E*, yield strength *R_p0.2_ and the resulting Orowan stress *σ_o_*. The Orowan stress is calculated by

(1) $$\sigma_{o}={}^{*}R_{p0.2,MC}-{}^{*}R_{p0.2,Ref}$$

where *R_p0.2,MC_ and *R_p0.2,Ref_ denote the compressive yield strength at 0.2% plastic strain of the MC-strengthened and the MC-free solid solution matrix of Co-15Re-5Cr (Ref9), respectively. Since the specimens were subjected to a preload of 100 MPa as mentioned beforehand, small strains within the elastic regime had already occurred before the strain measurement started. These small strains were estimated by dividing the preload by the respective average Young’s modulus of each alloy. Instead of shifting the stress–strain curves by the amount of the calculated strains, the offset was taken into account during the determination of *R_p0.2_* as the straight line starts at 0.2% minus the respective strain. Thus, using *R_p0.2_ instead of *R_p0.2_* for the denotation of the yield strength.

The stress–strain curves show some scattering for Ref9 in Figure 5a and TiC9 in Figure 5c whereas TaC9 and TaC11 in Figure 5b as well as TiC11 in Figure 5c are characterized by insignificant scattering since all three stress–strain curves overlap. The yield strength of the MC-strengthened Co-15Re-5Cr alloys is much higher than that of the MC-free alloy. However, this difference in yield strength decreases as the aging temperature rises from 900 °C to 1100 °C. Another point worth mentioning is that the yield strengths of both TaC- and TiC-strengthened Co-15Re-5Cr alloys are nearly the same, comparing the same aging treatment, which consequently leads to similar Orowan stresses. 

**Figure 6:**
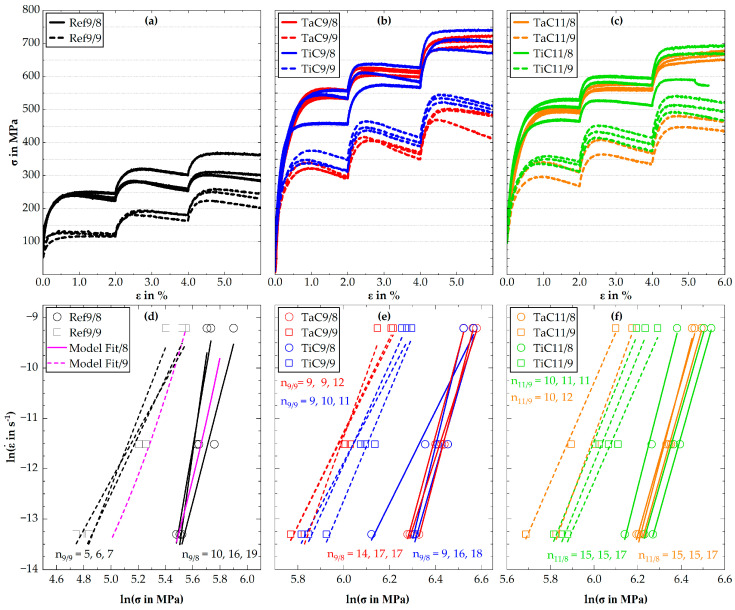
Strain-rate controlled creep curves (strain rates of 10^−6^ s^–1^, 10^−5^ s^–1^ and 10^−4^ s^–1^ corresponding to the strain steps of 0–2%, 2–4% and 4–6%) in (**a**–**c**) and stress exponents *n* in (**d**–**f**) of TaC9, TaC11, TiC9, TiC11 and Ref9. The abbreviation “slash with a subsequent 8 and 9” after the alloy denotation refers to the creep temperatures of 800 °C and 900 °C, respectively. The solid and dashed lines are the regression lines for the creep values at 800 °C and 900 °C with the same color scheme as presented in the legend. In addition, (**d**) depicts the calculated logarithmized strain rate over the creep stress obtained from the model proposed in Section 4.2 for the solution strengthened reference alloy at 800 °C (Model Fit/8) and 900 °C (Model Fit/9) illustrated also by a solid and dashed line, respectively.

The results from the compression creep study for the strain rates $\dot{\varepsilon }$ of 10^−6^ s^−1^, 10^−5^ s^−1^ and 10^−4^ s^−1^ at creep temperatures of 800 °C and 900 °C are illustrated in Figure 6. The MC-free alloy Co-15Re-5Cr in Figure 6a shows that the solid solution provides a creep strength of approx. 250 MPa and 125 MPa at 800 °C and 900 °C for 10^−6^ s^−1^, respectively. Increasing strain rates lead to a further increase in creep strength.

It is evident that at 900 °C and a strain rate of 10^−6^ s^−1^, a stationary creep state is present as an essentially constant stress occurs after a short phase of primary creep. However, at the higher strain rates, strain-induced softening takes place, manifesting itself as a decline of the creep stress *σ* after reaching its maximum value. At 800 °C, two out of the three creep curves exhibit this phenomenon already at 10^−6^ s^−1^. Strain-induced softening does essentially not appear in Figure 6b,c for TaC- and TiC-strengthened Co-15Re-5Cr at 800 °C, but is present for all curves at a testing temperature of 900 °C. This phenomenon is consistent for both MC-variants and independent of the aging temperature. The contribution of TaC and TiC to creep strength is almost identical. In the case of TaC-Co-15Re-5Cr, there is less experimental scattering than for the corresponding TiC-Co-15Re-5Cr alloy. However, both reach a creep stress of approx. 550 MPa at 800 °C and 10^−6^ s^−1^ when aged at 900 °C/15 h. This yields a contribution to creep strength due to particle strengthening of approx. 300 MPa, obtained by the difference in creep strength between MC-strengthened and MC-free alloy. For the creep temperature of 900 °C, both TaC9 and TiC9 range around similar creep stresses at 10^−6^ s^−1^ but TiC9 surpasses TaC9 at higher strain rates of 10^−5^ s^−1^ and 10^−4^ s^−1^ by approx. 40 MPa and 45 MPa, respectively. Taking the maximum creep stress value for the creep strength, a MC-strengthening contribution of approx. 205 MPa and 230 MPa for TaC9 and TiC9, respectively, is obtained at 10^−6^ s^−1^ when tested at 900 °C. Aging at 1100 °C/15 h leads to an overall decrease in creep strength, which affects TaC- and TiC-Co-15Re-5Cr at 800 °C similarly. The average creep stress value for TaC11 and TiC11 tested at 800 °C and 10^−6^ s^−1^ suggests a decrease in creep strength of approx. 45 MPa in comparison to aging at 900 °C/15 h, if the single outlier of TiC9 is not considered. The same observation cannot be made at 900 °C creep temperature as experimental scattering increases once more and TaC- and TiC-Co-15Re-5Cr reach similar creep stress values. 

An overview of all stress exponents $n={\left(\partial ln\left(\dot{\varepsilon }\right)/\partial ln\left(\sigma \right)\right)}_{T}$ (*T*: temperature) obtained for each creep curve is presented in Figure 6d–f. For this purpose, a linear regression of the logarithmized stress and strain rate values was performed, using the stationary or maximum stress values, respectively. In the case of Ref9, *n* ranges between 10 and 19 for an 800 °C creep temperature, whereas at 900 °C it is reduced to 5 to 7. As a consequence of the similarity regarding the absolute creep stress values of TaC- and TiC-Co-15Re-5Cr, the stress exponents do not significantly differ from each other. At a creep temperature of 800 °C, all stress exponents reach values between 14 and 18 (with a single outlier for TiC9) and are independent of the aging temperatures. A decrease in stress exponent to the range of 9 to 12 is observed when the creep temperature is increased to 900 °C.

**Figure 7:**
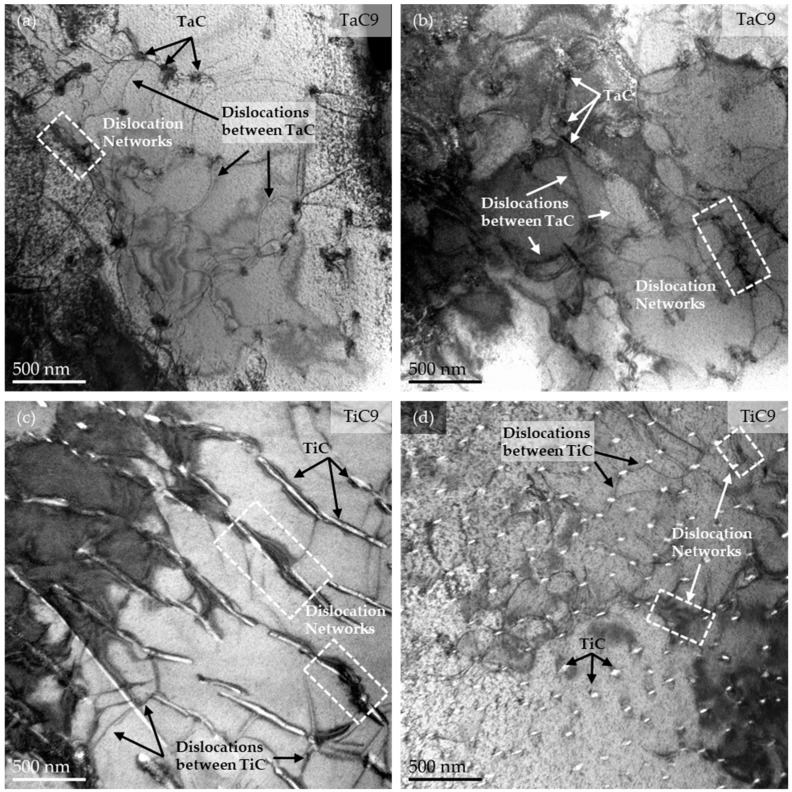
A selection of tilted TEM bright field images from representative areas illustrating the dislocation interaction with TaC and TiC, as well as the presence of dislocation networks after creep deformation at 800 °C for TaC9 in (**a**,**b**), as well as for TiC9 in (**c**,**d**).

The tilted TEM images in Figure 7 reveal that dislocations clearly interact with TaC and TiC. The samples were tilted several degrees towards {100} out of matrix [001]-zone axis orientation to approximate the two-beam reflection condition, hence facilitating the imaging of dislocations. Some dislocations are pinned on the particle–matrix interface and bow out as a result of dislocation propagation under creep (as seen in Figure 7a and also in Figure 3). This demonstrates that the carbide particles act as obstacles to dislocation motion during creep deformation, even though it is not possible to determine if the dislocations are generally pinned on the arrival or departure side of the particles. Other dislocations agglomerate into larger networks of self-interacting dislocations, as seen in the dashed box in Figure 7b.

## 4. Discussion

### 4.1. Precipitation, Morphology and Orientation Relationship

Figure 1 confirms the observation from [18] that TaC precipitates in Co-Re-based alloys appear either as fine globular or elongated nano-sized particles in the SEM images. The series of tilted TEM images in Figure 2a suggests that TaC exhibits a rod-like morphology that yields different cross-sections corresponding to the shapes (globular and elongated) observed here and in [18] (depending on the particle orientation relative to the cross-sectional area under consideration). However, Figure 3 clearly illustrates that a certain amount of TaC, indeed, can precipitate as equiaxed particles, as seen also in [16,17]. Analogously, the bar-like-shaped TiC can appear either as fine and nearly equiaxed or elongated particles in the SEM image. The fact that the size of the fine TaC and TiC particles, as seen in Figure 1, is comparable, suggests that the nucleation and growth behavior may be similar too. 

The P-SAED and ACOM results in Figure 4 suggest an orientation relationship of ($\bar{1}$20)_hcp-Co_ || ($\bar{1}$10)_TiC/TaC_ and [00$\bar{1}$]_hcp-Co_ || [112]_TiC/TaC_. It differs from that proposed in [18] for TaC with matching planes of (001)_hcp-Co_ || (01$\bar{2}$)_TaC_ and matching directions of [210]_hcp-Co_ || [100]_TaC_ as well as [010]_hcp-Co_ || [021]_TaC_. Note that the latter relationship was not determined experimentally but based on the edge-to-edge matching model [19] and the realization that (001)_hcp-Co_ || (01$\bar{2}$)_TaC_, [210]_hcp-Co_ || [100]_TaC_ exhibit particularly low misfits in planar as well as interatomic spacing. However, it has to be emphasized in this context that the P-SAED (TaC9 and TiC9) and ACOM (TiC9) measurements were performed for needle-like precipitates only in a single, well oriented grain of the respective bulk material. Therefore, it cannot be ruled out that other orientation relationships than the one determined here exist. One reason for differing orientation relationships could be the fcc-Co to hcp-Co phase transformation taking place in these alloys upon cooling from the solution heat treatment temperature. Hereby, differently oriented hcp-grains grow typically into one fcc-grain, but eventually only one dominant hcp-grain remains. As demonstrated by in-situ small angle scattering experiments [20], TaC precipitation already occurs during cooling once the fcc/hcp transformation starts. If TaC particles precipitate with an orientation relationship to a hcp-grain that is later overgrown by another one, the particles do not reorientate themselves to the final grain orientation. Thus, it may be possible to observe different orientations of TaC in hcp-Co if different particles are contemplated. Nevertheless, the fact that the same orientation relationship was found here for the TiC and TaC particles indicates a preferred orientation relationship between hcp-Co and MC-carbides, which have a NaCl-type crystal structure. Apparently, the different lattice constants of TaC and TiC do not lead to different orientation relationships.

### 4.2. Compression Tests at Ambient and Elevated Temperatures, Particle–Dislocation Interaction

The compression tests at room temperature revealed a surprisingly low compressive Young’s modulus for the MC-strengthened and MC-free Co-15Re-5Cr alloys. Due to the general correlation between the Young’s modulus and the melting temperature, the authors expected values at least comparable to that of pure Co, which was determined as 204 GPa in [21] by a high-accuracy set-up based on laser Doppler vibrometry [22]. The binary systems of Co-Re and Co-Cr suggest melting temperatures for a Re-content of 15% and Cr-content of 5% at around 1560 °C and 1495 °C, respectively, being either higher than the one of pure Co at 1495 °C or essentially unchanged [7]. The slight increase in the Young’s modulus observed for the MC-strengthened alloys is in good agreement with the fact that the Young’s modulus of (nearly) stochiometric TaC_0.97–1.0_ and TiC_0.95–1.0_ is much higher than the one of the matrix, ranging from 537 GPa to 567 GPa and from 436 GPa to 460 GPa, respectively [23]. Thus, even small amounts of hard precipitates may contribute to a slightly higher macroscopic stiffness of the particle-containing alloys compared to their particle-free counterpart. However, the comparatively low Young’s modulus in general gave rise to the suspicion that there might be a systematic error during the compression test. In fact, focused contemplation of the low strain region revealed that the compression plates were not completely plane parallel to the front faces of the specimens at the beginning of the compression test. The lack of plane parallelism leads to the phenomenon that the specimen’s front planes are already in contact with the compression plates on one side but not yet on the other side. Apparently, the strain was measured with the GOM camera from the side, which was in contact with the compression plates from the beginning. This led to an overestimation of the strain because the camera was focused on the area that deformed first under low forces and, consequently, resulted in a lower Young’s modulus. Nevertheless, the estimation of the Orowan stress by Equation (1) is still considered a valid approach since all stress–strain curves are similarly affected and the respective yield strength values were determined at identical offset strains relative to the experimentally determined elastic line.

The creep experiments on Co-15Re-5Cr at 800 °C revealed a rather high stress exponent *n* between 10 and 19. Often, stress exponents of 3 to 5 are observed in solid solutions. Stress exponents of about 3 are rationalized assuming (i) viscous movement of the dislocation with velocity v~σ (*σ*: applied stress) and (ii) a dislocation density $\rho \sim \sigma^{2}$ so that a shear rate $\dot{\gamma }=\rho \cdot v\cdot b\sim \sigma^{3}$ results (*b*: Burgers vector). Hereby, the drag of the dissolved atoms by the moving dislocations is seen as a cause for the linear relation between stress and dislocation velocity [24]. However, if the solute atoms are sufficiently immobile and/ or the dislocation velocity is too high, it is neither possible to drag the dissolved atoms along nor to circumvent them by climbing. Then, they pose an energy barrier that has to be overcome by thermal activation, provided the applied stress *σ* is below the peak stress *σ_p_* required to overcome the barrier in the absence of thermal activation (i.e., at 0 K). In this case, the strain rate is given by

(2) $$\dot{\varepsilon }={\dot{\varepsilon }}_{0}\cdot e^{\left(-\frac{E_{a}}{k_{B}T}\right)}$$

where ${\dot{\varepsilon }}_{0}$ is a reference strain rate, *k_B_* the Boltzmann constant and *E_a_* the stress dependent activation energy. 

As shown by Kocks et al. [25], the latter term generally has the form

(3) $$E_{a}=E_{0}\cdot {\left(1-{\left(\frac{\sigma }{\sigma_{p}}\right)}^{p}\right)}^{q}$$

with 0 < *p* ≤ 1 and 1 ≤ *q* ≤ 2. Hereby, *E_0_* is the activation energy when the stress is zero. Neglecting the weak stress dependence of ${\dot{\varepsilon }}_{0}$, which contains the stress dependent dislocation density, the stress exponent is then given by

(4) $$n={\left(\frac{\partial ln\left(\dot{\varepsilon }\right)}{\partial ln\left(\sigma \right)}\right)}_{T}=\frac{p\cdot q\cdot E_{0}}{k_{B}T}\cdot {\left(1-{\left(\frac{\sigma }{\sigma_{p}}\right)}^{p}\right)}^{\left(q-1\right)}\cdot {\left(\frac{\sigma }{\sigma_{p}}\right)}^{p}$$

demonstrating that stress exponents *n* » 3 result if *E_0_* » *k_B_T*. Thus, the high stress exponent observed here at 800 °C clearly indicates that the Re-atoms act as strong obstacles to dislocation motion. Apparently, their mobility is still too low to be dragged along by the dislocations, so they have to be overcome by thermal activation.

At 900 °C, the stress exponent of Ref9 drops considerably to an average of 6. To analyze this, we use an energy barrier as suggested by Varvenne et al. [26] for solid solutions of the form

(5) $$E_{a}=E_{0}\cdot {\left(1-\left(\frac{\sigma }{\sigma_{p}}\right)\right)}^{3/2}$$

The quotient *m* between the stress exponents at two temperatures is then given by

(6) $$m=\frac{n\left(T_{1}\right)}{n\left(T_{2}\right)}=\frac{T_{2}}{T_{1}}\cdot \frac{{\left(1-\frac{\sigma \left(T_{1}\right)}{\sigma_{p}}\right)}^{1/2}\cdot \left(\frac{\sigma \left(T_{1}\right)}{\sigma_{p}}\right)}{{\left(1-\frac{\sigma \left(T_{2}\right)}{\sigma_{p}}\right)}^{1/2}\cdot \left(\frac{\sigma \left(T_{2}\right)}{\sigma_{p}}\right)}$$

At a strain rate of 10^−6^ s^−1^, the flow stresses are about 250 MPa and 125 MPa at 800 °C and 900 °C, respectively. The peak stress *σ_p_* needed to overcome the energy barrier associated with the dissolved Re-atoms is unknown. However, compression tests at ambient temperature, presented in Figure 5, revealed a yield strength of 740 MPa for Ref9. Since dislocation densities are typically low in the as-cast and solution treated state with subsequent heat treatment at 900 °C for 15 h and the grain size is relatively large (about 70 µm), the yield strength can be essentially attributed to solid solution strengthening, so that it is reasonable to assume a value for *σ_p_* in excess of 740 MPa. Fortunately, *m* only weakly depends on *σ_p_*. For the two above-mentioned temperatures (*T_1_* = 1073 K, *T_2_* = 1173 K), it varies between 2.0 and 2.1 for values of *σ_p_* between 740 MPa and 2000 MPa. Thus, the observed drop in the stress exponent from an average value of 15 at 800 °C to an average of 6 at 900 °C, leading to *m* = 2.5, is consistent with the view that the creep strength is controlled by the energy barrier due to the Re-atoms, which is overcome by thermal activation. In this context, it has to be pointed out again that the creep curves do not display a perfect steady state but some degree of softening. As softening proceeds with ongoing strain irrespective of the applied strain rate, it affects the flow strength at a strain rate of 10^−5^ s^−1^ more than at 10^−6^ s^−1^ and even more so at 10^−4^ s^−1^ since the strain is increasing in this order. This, in turn, leads to an overestimation of the stress exponent, which ideally should be determined at a constant degree of strain-induced softening. 

This effect can be well seen when the creep curves for Ref9 at 800 °C are inspected. Here, strain-induced softening is the least pronounced for the curve exhibiting the highest strength. Consequently, the lowest stress exponent of 10 was obtained for this curve. Noticing, furthermore, that strain-induced softening of Ref9 is slightly more noticeable at 800 °C than at 900 °C, it understandable why the experimentally determined *m*-value is somewhat higher than the calculated one.

Given the available data, it is not possible to determine *E_0_* and *σ_p_* precisely. However, since *E_0_* and *σ_p_* influence the resulting creep strength and stress exponent in different ways (most notably, the stress exponent is increasing with increasing *E_0_* (at constant *σ_p_*) but decreasing with increasing *σ_p_* (at constant *E_0_*) because the energy barrier is becoming wider in the first case but narrower in the second), they cannot be chosen arbitrarily. In fact, to reasonably reproduce the experimentally observed creep strength and stress exponents at 800 °C and 900 °C, *E_0_*(0 K) ≈ 4.9eV and *σ_p_*(0 K) ≈ 1700 MPa are required, assuming ${\dot{\varepsilon }}_{0}$ = 1∙10^5^ s^−1^ as suggested in [26] when considering solid solution strengthening. Note that *E_0_* and *σ_p_* are proportional to the dislocation line energy, which in turn is proportional to the shear modulus. Thus, *E_0_* and *σ_p_* are temperature dependent. To reflect this, the quantities at temperature *T* were calculated by $E_{0}\left(T\right)=E_{0}\left(0K\right)\cdot G(T)/G(0K)$ and $\sigma_{p}\left(T\right)=\sigma_{p}\left(0K\right)\cdot G(T)/G(0K)$ using *dG/dT* = −26 MPa/K [27] and *G*(293 K) = 77.3 GPa, obtained from *E* = 204 GPa as Young’s modulus at room temperature [21] and Poisson´s ratio *ν* = 0.32 [28]. As a result, *E_0_* = 3.29 eV, *σ_p_* = 1141 MPa and *E_0_* = 3.14 eV, *σ_p_* = 1089 MPa were obtained at 800 °C and 900 °C, respectively. With these parameters, the strain rate according to Equation (2) is plotted in Figure 6d as a function of applied stress, showing satisfactory agreement with the experimental data. In [29], *E_0_* = 1.76 eV was obtained for the equiatomic high entropy alloy CrMnFeCoNi at 800 °C with 318 MPa as contribution by solid solution strengthening to *σ_p_*. Even though there are differences in the performed analysis, notably *p* = *q* = 1 as well as a contribution from work hardening was assumed in [29], the comparison shows again that the Re-atoms are remarkably strong barriers to dislocation motion in Co-Re-based alloys.

As mentioned already, the strengthening associated with the TaC- and TiC-particles is about 300 MPa at 800 °C in both cases. Compression tests at room temperature revealed an increase in yield strength by the same amount. Due to the inability of the dislocations to climb and the dissimilarity of the crystal structure between matrix and particles, it is likely that the particles are bypassed by the Orowan mechanism at ambient temperature. Despite the additional possibility to overcome the particles by climbing at 800 °C, the comparable strengthening contribution of the particles at both temperatures shows that the particles are still strong obstacles to dislocation motion at 800 °C. It means that climbing over the particles and/ or dislocation detachment from their backside must be very difficult. This is consistent with the TEM-observations after creep deformation, which show that the dislocations are frequently pinned at the particles. To gain further insight, the detachment process is analyzed next. The basic idea is that the dislocation may be able to relax some of its line energy at the particle–matrix interface, thus becoming stuck there [30,31,32,33]. If that process is rate controlling, the relaxation factor $k=T_{p}/T_{m}$ (*T_p_*, *T_m_*: dislocation line tension at the particle and matrix, respectively) can be calculated from the stress exponent *n* by [33]

(7) k=1−2kBT3Gb2r·n1−σσd12·σσd23

(*G*: shear modulus, *b*: Burgers vector, *r*: particle radius). Hereby, *σ_d_* is the athermal detachment stress, i.e., the stress required for the dislocation to detach itself from the particle in the absence of thermal activation. It is related to the Orowan stress *σ_O_* by [32]

(8) $$\sigma_{d}=\sigma_{O}\sqrt{1-k^{2}}$$

In [18] lattice parameters were measured for Co-17Re-1.2Ta-1.2C after ST plus heat treatment at 900 °C. With these values, *b* = 2.56 × 10^−10^ m results, assuming that dislocation slip takes place mostly along the basal plane of the hexagonal lattice. Furthermore, shear modulus and particle radius are approximated by *G* = 57 GPa at 800 °C (obtained from elastic constants stated above) for hcp-Co and *r* = 20 nm. With *n_TaC9/8_* ≈ 16 at 800 °C, *k* ≈ 0.92–0.97 results for any value of *σ*/*σ_d_* between 0.1 and 0.99. Even for the lowest value of *k*, *σ_d_* would be just 39% of the Orowan stress, which by itself is an upper bound for the strengthening contribution of the particles if dislocation detachment were the rate controlling strengthening mechanism. This is in contrast to the experimental observation that the particle strengthening effect at 800 °C is very similar to that at ambient temperature, i.e., close to the Orowan stress. Consequently, dislocation detachment cannot be the rate controlling mechanism, even though it may contribute to a certain extent. (Note that Equation (7) was derived for spherical particles. Their radius r enters Equation (7) because it determines the curvature of the particle surface at the location where the dislocation is about to detach itself from the particle. For the elongated particles observed here, the actual curvature depends on the orientation of the particle relative to the intersecting glide plane and the direction of dislocation motion in that glide plane. Using the short axis of the particles, i.e., *r* = 20 nm, here overestimated the average curvature the dislocation experiences during detachment. Consequently, *k* and *σ_d_* are somewhat under- and overestimated, respectively. This substantiates the drawn conclusion that dislocation detachment cannot be rate controlling even more so.) Thus, it must be concluded that dislocation climb over the particles is the decisive mechanism retarding dislocation motion under the creep conditions investigated here. In this context, the high aspect ratio of the particles has to be noted. This is a far more effective particle shape compared to an equiaxed one to hinder dislocation climb because the dislocation must climb farther out of its glide plane unless the long axis of the particles is oriented essentially parallel to the glide plane. This requires more vacancies, i.e., more time. Furthermore, a dislocation will thread over and under neighboring particles, so that a certain amount of additional dislocation line length is needed to surmount the particles. This leads to a threshold stress that has to be exceeded. Otherwise, the extra energy needed to create additional dislocation line length cannot be provided by the applied stress. Rösler and Arzt [34] calculated the geometry of a climbing dislocation past a cubic particle by requiring a constant chemical potential of the vacancies along the climbing dislocation line. They found that the incremental increase in dislocation line length *dl* per incremental advance in glide direction *dy* increases as the dislocation climbs out of the glide plane, so that the highest stress required to advance the dislocation further is reached at the top of the particle. The resulting threshold stress *σ_th_* is given for small particle volume fractions by

(9) $$\sigma_{th}=\sigma_{O}\cdot \frac{h}{\lambda }$$

when the side face of the cubic particle is inclined by 45° to the glide plane (*h*: particle height, i.e., distance the dislocation has to climb out of its glide plane; *2λ*: interparticle spacing). Similar expressions were also found in other investigations [35,36]. This once again illustrates that elongated particles can be very effective barriers to dislocation climb because, given the overall particle size, the distance a dislocation has to climb out of its glide plane to surmount the particle is generally rather large. Note also that the threshold stress reaches the Orowan stress as soon as *h*/*λ* reaches a critical value, which depends on the respective particle geometry. In this case, climbing is no longer possible and the particles have to be overcome by the Orowan mechanism even at elevated temperatures. In fact, the strength difference between particle-strengthened and particle-free material at 800 °C is close to the value obtained at room temperature by compression testing. This suggests that the threshold stress due to dislocation climb is a significant fraction of the Orowan stress, so that the remaining effective stress $\sigma_{eff}=\sigma -\sigma_{th}-\sigma_{matrix}$ (*σ_matrix_*: stress required to creep deform the particle-free matrix) is not sufficient to drive the dislocations sufficiently fast past the particles by climb. In consequence, the Orowan mechanism is the only remaining option, resulting in comparable strengthening at 800 °C and room temperature. At 900 °C, the strengthening effect of the particles is less than at 800 °C, yet, still significant. At a strain rate of 10^−6^ s^−1^, it is approximately 205 MPa to 230 MPa compared to 300 MPa at 800 °C. This suggests that diffusion is now fast enough for dislocation climb to occur so that the strengthening contribution of the particles diminishes below the Orowan stress. 

At 900 °C creep temperature, the obtained stress exponent is consistently higher for the particle-strengthened material compared to the particle-free one. However, strain-induced softening is also consistently more pronounced in the former case. This may be the main reason for the observed difference. In fact, if the stress jump from the end of the creep curve at 1.67 × 10^−6^ s^−1^ to the maximum stress at the following strain rate (i.e., 10^−5^ s^−1^) is used to determine the stress exponent, *n* = 4, 4, 4, *n* = 5, 5, 5 and *n* = 5, 5, 6 are obtained for Ref9, TaC9 and TiC9, respectively. This suggests that there is little influence of the particles on the stress exponent, so that it is essentially determined by the matrix.

Finally, a comment on the threshold stress Equation (9) is in order. The basic assumption is that the dislocation climbs in a zig-zag pattern over neighboring particles. McLean [37] suggested that a dislocation may surpass neighboring particles in a cooperative way, whereby a dislocation segment climbing in a specific direction not only overcomes one but several particles. This reduces the amount of extra line length needed, i.e., the threshold stress. On the other hand, more vacancies, i.e., a faster flux of vacancies, are required. Thus, it might be that the threshold stress for dislocation climb is decreasing with increasing temperature (i.e., faster flux of vacancies) as the climb geometry changes from a zig-zag one to a more cooperative one. Also, from this point of view, it is understandable that the strengthening effect of the particles diminishes from 800 °C to 900 °C. This thought also illustrated that so-called threshold-stresses in creep are usually not “hard” barriers. Instead, the material finds a way around it in one way or another as time and temperature increase.

## 5. Conclusions

TaC and TiC in Co-Re-Cr with a composition of Co-15Re-5Cr-1.8Ta-1.8C and Co-15Re-5Cr-1.8Ti-1.8C pose a promising option for creep application as both tend to precipitate as nano-sized particles, which evidently interact with the dislocations and equally provide additional creep strength at 800 °C in the range of the Orowan stress (determined at RT) and slightly less at 900 °C, in comparison to the particle-free matrix. The analysis of the creep data suggests that the detachment process is not rate controlling for neither TaC- nor TiC-Co-Re-Cr. Instead, it appears that dislocation climb is the governing rate controlling mechanism. High ratios of climbing height to interparticle spacing may emerge when the long axis of the elongated particles is oriented non-parallel to the glide plane, causing a high threshold stress and slowing down the creep rate as more vacancies are needed to surmount the particle. Furthermore, the solid solution strengthened Co-15Re-5Cr matrix significantly contributes 250 MPa (at 10^−6^ s^−1^) to the creep strength at 800 °C as a result of the strong solid solution strengthening effect of the Re-atoms being not sufficiently mobile to be dragged along by the moving dislocations. The drop in creep strength to 125 MPa (at 10^−6^ s^−1^) and stress exponent at 900 °C is consistent with the view that the Re-atoms are overcome by thermal activation in that temperature regime. Furthermore, TEM analysis revealed an orientation relationship between matrix and the TaC/ TiC particles of the type ($\bar{1}$20)_hcp-Co_ || ($\bar{1}$10)_TiC/TaC_ and [00$\bar{1}$]_hcp-Co_ || [112]_TiC/TaC_.

As strengthening by the dissolved Re-atoms and the MC-particles turned out to be very effective, one can finally raise the question, if these strengthening contributions can be further enhanced. Given the complete miscibility of Co and Re, an obvious choice is to further increase the Re-content. This measure would have to be balanced by addition of fcc-stabilizing elements to maintain the fcc high temperature phase for dissolution of the MC-precipitates. As far as particle strengthening by the MC-carbides is concerned, utilizing Ta and Ti simultaneously in Co-Re-Cr-Ta-Ti-C alloys may allow for an increase in the combined solubility of the MC-forming elements and, by that, the amount of MC-precipitates beyond the limit observed in Co-Re-Cr-Ta-C and Co-Re-Cr-Ti-C alloys. Even higher amounts of the MC-forming elements may be possible by additive manufacturing, allowing for substantially supersaturated solid solutions after solidification due to the fast cooling rates. Then solution heat treatment would no longer be required and also not useful, which would eliminate the need for a fcc high-temperature phase. However, as far as high-temperature applications are concerned, insufficient grain size might turn out as an issue to be tackled when this processing route is used.

## Figures and Tables

**Table 1:** Overview of the average values of the compressive Young’s modulus *E*, yield strength *R_p0.2_ and Orowan stress *σ_o_* for TaC9, TaC11, TiC9, TiC11 and Ref9 derived from compression tests at room temperature, as seen in Figure 5.

	Young’s modulus *E* in GPa	Yield strength *R_p0.2_ in MPa	Orowan stress *σ_o_* in MPa
**Ref9**	135	740	-
**TaC9**	154	1027	287
**TaC11**	154	941	201
**TiC9**	167	1049	309
**TiC11**	154	959	219

## Data Availability

The data presented in this study are available on request from the corresponding author.
